# The Role of Online Support, Caregiving, and Gender in Preventative Cancer Genetic Testing Participation: Cross-Sectional Study From a National Study

**DOI:** 10.2196/67650

**Published:** 2025-06-04

**Authors:** Lavlin Agrawal, Richelle Oakley DaSouza, Pavankumar Mulgund, Pankaj Chaudhary

**Affiliations:** 1 Department of Business Information Systems and Analytics, Willie A. Deese College of Business and Economics, North Carolina A&T State University, 1601 E. Market Street, 337 Merrick Hall, Greensboro, NC, 27411, United States, 1 7166508654; 2 Department of Management Information Systems, Fogelman College of Business & Economics, University of Memphis, Memphis, TN, United States

**Keywords:** genetic testing, cancer, health belief model, caregiver status, online social support, gender differences

## Abstract

**Background:**

Despite its potential to predict and detect early cancer risks, genetic testing remains underused by the public. This study, guided by the health belief model (HBM), examined key factors influencing an individual’s willingness to undergo genetic testing for cancer, with a particular focus on gender, caregiver status, and participation in online social support groups.

**Objective:**

This study aimed to explore the factors that can influence the individual’s decision to undergo preventative genetic testing for cancer so that more informed action can be taken to encourage the individuals to engage in preventative health behavior.

**Methods:**

This study uses data collected from the 2020 Health Information National Trends Survey (HINTS 5 Cycle 4), which included 2947 respondents representing 199,510,996 US adults aged 18 years and older. Multivariable logistic regression and survey-weighted generalized linear models were applied to examine the relationship between cancer genetic testing and caregiver status, participation in online support groups, gender, and constructs associated with the HBM, while controlling for sociodemographic and health-related characteristics.

**Results:**

Our findings show that women are more likely to undergo cancer genetic testing, with gender moderating the influence of perceived susceptibility (*β*=2.54, *P*=.03) and severity (*β*=0.94, *P*<.050) on testing decisions. In line with the HBM, perceived benefits (*β*=0.19, *P*=.03) and cues to action (*β*=2.86, *P*<.001) increase the likelihood of testing. Results also show that caregivers of patients with cancer (*β*=1.25, *P=*.04) and those actively participating in online health support groups (*β*=0.47, *P*=.04) are also more likely to engage in cancer genetic testing.

**Conclusions:**

Cancer remains a significant health challenge in the United States, with 1.8 million new cases and 606,520 deaths annually. Early detection is vital for treatment success. This study investigates factors influencing the decision to undergo genetic testing for cancer. The examination of caregiver status and online support groups as influencing factors, along with the HBM, provided a significant theoretical contribution to the health care research domain. Results indicated that caregivers and men should be directly targeted with messaging on genetic cancer screening as a proactive health behavior. Additionally, online support groups can promote early detection and encourage participation in genetic testing. Future research should further explore implementing proactive outreach strategies to encourage wider adoption of genetic testing for cancer.

## Introduction

### Overview

Cancer remains one of the most urgent health challenges in the United States, with approximately 2 million new cases and 611,000 deaths projected to occur in the United States in 2024 [1]. These figures highlight the importance of early detection and timely intervention, which are critical for improving treatment outcomes and reducing cancer-related mortality [2]. Early detection, particularly through predictive methods, enables health care providers to identify cancers at earlier stages, where treatment options can significantly improve the chances of successful outcomes. Consequently, the development and widespread adoption of effective screening strategies have become paramount in the fight against cancer.

Among these strategies, genetic testing has emerged as a pivotal tool in cancer prevention and early detection [3]. By analyzing an individual’s DNA, genetic testing can identify mutations that may predispose individuals to various forms of cancer [4]. This predictive capability empowers individuals with knowledge that allows them to take preventive actions or pursue early treatments. As medical science and technology continue to advance, the scope of genetic testing has expanded to detect a wide range of inherited disorders, making it an indispensable approach for reducing cancer risk [5,6].

The health belief model (HBM) offers a valuable framework to understand why individuals engage in preventive behaviors like genetic testing. The HBM suggests that individuals’ health behaviors are shaped by their perceptions of susceptibility, severity, and the benefits of action [7]. This model has been widely applied to the study of various preventive health actions, including vaccinations for H1N1 [8] and COVID-19 [9], as well as screening for conditions like diabetes [10] and several types of cancer [11-14]. Alongside the HBM, three key factors—caregiver status, participation in online social support groups, and gender—are likely to influence preventive behaviors related to genetic testing.

Caregivers, who are often family members, gain a deeply personal and intimate understanding of cancer’s devastating impact. Due to shared genetic ties and family history, these caregivers frequently face a higher risk of developing cancer themselves [15,16]. Their close connection to the disease makes them a critical demographic for studying behaviors related to genetic testing. Given their heightened risk and firsthand exposure to cancer, caregivers may be particularly motivated to pursue genetic testing as a preventive measure.

In addition to caregiver status, online social support groups have been shown to play a crucial role in promoting preventive behaviors. Hwang et al [17] demonstrated that participation in online peer support groups significantly increased motivation for colorectal cancer screening, reinforcing participants’ belief in the effectiveness of early detection. Lastly, gender has a profound influence on preventive health actions. Research indicates that women tend to be more proactive in engaging in preventive health behaviors [18-20]. For instance, women with low social support are less likely to participate in breast cancer screening, further illustrating the interplay between social support and gender in health decision-making behaviors [21].

The remainder of this paper is organized as follows. First, we provide an overview of the literature on genetic testing and the HBM. Next, we develop hypotheses and propose our research model. We then outline the research methodology and present the results. Following this, we discuss the findings, exploring both theoretical and practical implications. Finally, we conclude with a summary of the key insights gained from this research and address its limitations.

### Background

Advancements in identifying gene mutations have made genetic testing a crucial tool in reducing illness and death by enabling early detection and preventive measures [5]. Predictive genetic testing, which assesses an individual’s risk of developing diseases like cancer, focuses on identifying genetic mutations linked to disease susceptibility [22]. However, challenges persist in diagnosing hereditary breast and ovarian cancers, particularly in terms of genetic literacy and interpreting test results [23]. Nelson et al [24] emphasize the critical role of genetic counseling in BRCA-related cancer testing, highlighting the importance of providing informed decision-making support to patients.

Prior research also suggests that people with a family history of cancer often overestimate their risk. Their decisions about genetic testing are often influenced more by their subjective perceptions of vulnerability than by objective data [25]. This cognitive bias can lead to increased anxiety. It may cause individuals to rush into testing without enough information or avoid testing out of fear of the results. Despite these misperceptions, genetic testing consistently offers significant benefits, regardless of the outcome. Whether the results confirm a genetic predisposition or provide reassurance, testing allows individuals to make informed health decisions, take preventive measures, and access appropriate counseling and support services [26]. This highlights the importance of education and counseling in helping individuals to more accurately interpret genetic risks and use the information to make effective health care choices.

Caregivers of patients with cancer often play a pivotal role in health care decision-making, including decisions about genetic testing [27]. As primary support figures, caregivers are frequently involved in gathering health-related information, navigating complex medical choices, and encouraging preventative behaviors. Social support groups, both in-person and online, have been shown to provide critical informational and emotional support during these processes [28]. With the rise of online communities, individuals considering genetic testing now have greater access to peer support and shared experiences. Silence [29] demonstrated that online cancer communities facilitate advice-seeking and information-sharing, creating a valuable space for individuals to navigate genetic testing options. Similarly, Ruco et al [30] found that online social interactions significantly increase participation in cancer screening, highlighting the potential of these platforms to influence health-related behaviors. Given their influence in health decisions and the growing prevalence of online support networks, understanding the combined impact of caregiver status and participation in online social support groups is essential for accurately capturing the social and behavioral drivers behind genetic testing decisions.

The HBM is frequently used to examine health behaviors by considering factors such as perceived susceptibility, severity, cues to action, benefits, barriers, and demographic variables [7]. Bunn et al [31] and Hartman [32] successfully applied the HBM to predict decisions about preventive screenings, like colon cancer screening and mammography. The HBM provides a robust framework for understanding and influencing health-related decision-making, particularly in the context of preventive interventions such as genetic testing.

### Theoretical Model

#### Health Belief Model—Perceived Susceptibility

Perceived susceptibility is an individual’s belief about their likelihood of experiencing a health condition and is a key driver of health-related behavior according to the HBM [33,34]. Research consistently demonstrates that higher perceived susceptibility significantly influences medical decisions. For instance, Champion and Skinner [35] found that women with greater perceived susceptibility to breast cancer were more likely to undergo mammography. Similarly, Irigoyen-Camacho et al [36] observed that older adults with heightened perceived susceptibility to COVID-19 engaged more in preventive behaviors. Meta-analyses further confirm the strong correlation between perceived susceptibility and health behavior change [37,38]. Thus, we hypothesize the following:

Hypothesis H1a: Perceived susceptibility positively influences an individual’s decision to undergo genetic testing for cancer.

Further, the impact of perceived susceptibility varies by gender. Studies suggest that women generally perceive greater health risks and, therefore, are more likely to adopt health-promoting behaviors, such as regular screenings and healthier lifestyles [39]. For example, Lisha et al [40] found that women with similar levels of physical activity as men had lower alcohol consumption. Based on this, we hypothesize the following:

Hypothesis H1b: Gender moderates the relationship between perceived susceptibility and an individual’s decision to undergo genetic testing for cancer.

#### Health Belief Model—Perceived Severity

Perceived severity refers to an individual’s belief about the seriousness of contracting an illness [34]. According to the HBM, higher perceived severity increases motivation to engage in health-promoting behaviors [35]. Studies by Witte and Allen [41] and Brewer et al [37] show that individuals who perceive health threats as severe are more likely to adopt preventive measures, such as vaccination. Similarly, Irigoyen-Camacho et al [36] found that the perceived severity of COVID-19 significantly influenced compliance with stay-at-home guidelines. He et al [42] also reported that individuals with high perceived severity of colorectal cancer were more likely to undergo colonoscopy. These findings highlight the pivotal role of perceived severity in driving medical action. Thus, we hypothesize the following:

Hypothesis H2a: Perceived severity positively influences an individual’s decision to undergo genetic testing for cancer.

Further, extant research found that women perceive risks higher and engage in more preventive health behaviors [43]. Women generally report higher levels of perceived severity regarding health issues compared to men, and this heightened perception is linked to greater engagement in preventive health behaviors [39]. Sattler et al [44] also demonstrated that women were more likely than men to perceive the severity of health threats such as COVID-19, which translated into a higher likelihood of adopting recommended health behaviors. Thus, we extend this notion and hypothesize the following:

Hypothesis H2b: Gender moderates the relationship between perceived severity and an individual’s decision to undergo genetic testing for cancer.

#### Health Belief Model—Perceived Benefit

Perceived benefits refer to an individual’s belief in the positive outcomes of a health action, and they play a crucial role in motivating behavior change [35]. For instance, individuals are more likely to engage in physical activity if they believe it will lead to significant health improvements [45]. Similarly, Chen et al [46] found that perceived benefits significantly impact decisions to get vaccinated. Chen et al [47] further confirmed the association between perceived benefits and preventive behaviors. Bosompra et al [48] found that perceived benefits significantly impacted decisions to undergo genetic testing for cancer susceptibility. Based on this, we hypothesize the following:

Hypothesis H3: Perceived benefits positively influence an individual’s decision to undergo genetic testing for cancer.

#### Health Belief Model—Perceived Barrier

Perceived barriers refer to an individual’s assessment of obstacles that hinder the adoption of health-related behaviors [34]. Research consistently shows that these barriers negatively impact medical decision-making. According to the HBM, barriers may include factors such as cost, time, inconvenience, and fear of adverse outcomes [35]. A systematic review by Al-Noumani et al [49] identifies perceived barriers as a key predictor of poor adherence to health behavior changes in chronic conditions. Similarly, studies demonstrate that perceived barriers influence compliance with COVID-19 preventive measures over time [50,51]. Building upon these findings, we hypothesize the following:

Hypothesis H4: Perceived barriers negatively influence an individual’s decision to undergo genetic testing for cancer.

#### Health Belief Model—Cues to Action

Cues to action are stimuli that prompt individuals to engage in health-promoting behaviors and are crucial for motivating medical action [35]. In the HBM, cues can be internal, such as experiencing symptoms, or external, like advice from others or health campaigns. Carpenter [52] found that both internal cues, like symptoms, and external cues, such as media messages, significantly increase the likelihood of seeking medical care. Similarly, Glanz et al [53] showed that health communication campaigns effectively acted as external cues, leading to higher vaccination and screening rates. Based on this, we hypothesize the following:

Hypothesis H5: Cues to action positively influence an individual’s decision to undergo genetic testing for cancer.

### Caregiving

A caregiver provides care and support to someone with health-related needs due to chronic illness, disability, or aging [54]. When a family member is diagnosed with cancer, caregivers are deeply involved in diagnosis, treatment, and survivorship care [55]. During this time, they interact closely with health care providers, gather medical information, and witness their loved one’s experiences. The Center for Disease Control encourages caregivers to practice self-care and engage in preventive health care [56]. Research shows that spousal caregivers are more likely to undergo cancer screenings [57,58], with evidence of increased screening for stomach, breast, and cervical cancer among caregivers [59]. Additionally, caregivers are more likely to adopt health-promoting behaviors [60]. Extending this idea to genetic testing, we hypothesize the following:

Hypothesis H6: Caregiving positively influences an individual’s decision to undergo genetic testing for cancer.

### Online Social Support

Online social support groups provide a platform for individuals to share experiences and receive encouragement from others facing similar health challenges. This support fosters motivation and adherence to health goals. Participants often feel more committed to their health plans when they receive positive feedback and peer encouragement [61]. These groups also offer emotional support, reducing stress and anxiety, leading to better adherence to health-promoting behaviors and overall well-being [62]. Additionally, personalized advice from peers who have faced similar issues can be more practical and relevant than generic information, resulting in more effective health behavior changes [63]. A systematic review further supports the effectiveness of social media tools in delivering interventions for cancer prevention and management [64,65]. Based on these insights, we hypothesize the following:

Hypothesis H7: Participation in online health communities positively influences an individual’s decision to undergo genetic testing for cancer.

**Figure 1.**
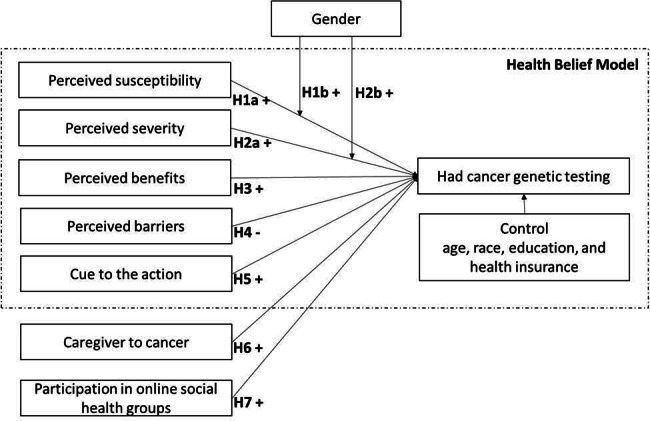
Research model.

Based on these hypotheses, our proposed research model is presented in Figure 1.

## Methods

### Data

We used data from the 2020 National Cancer Institute Health Information National Trends Survey (HINTS 5 Cycle 4) survey, which is publicly available on the HINTS website [66]. Table 1 presents the correlations between the principal variables with survey weights. High correlations can indicate multicollinearity, but all correlations fall below the threshold of 0.5 [67], so multicollinearity was not an issue. The table also includes the means and SDs for the key variables.

**Table 1.** Correlation matrix.

	Mean	SD	PSUS^a^	PSEV^b^	PBEN^c^	CUES^d^	CC^e^	OSS^f^
PSUS	0.732	0.665	1.000					
PSEV	2.509	0.951	0.010	1.000				
PBEN	9.873	1.585	0.060	0.010	1.000			
CUES	0.438	0.704	0.160	−0.120	0.100	1.000		
CC	0.019	0.369	0.030	0.030	−0.090	0.020	1.000	
OSS	0.266	0.746	−0.020	0.040	0.060	0.040	0.010	1.000

a PSUS: Perceived susceptibility.

b PSEV: Perceived severity.

c PBEN: Perceived benefit.

d CUES: Cues to action.

e CC: Caregiving cancer.

f OSS: Online social support.

### Measurements

The complete set of questionnaires for the dependent, independent, and control variables can be found in Multimedia Appendix 1. Although many variables are measured using multi-item scales, some are captured through single-item measures. The use of single-item measures is deemed appropriate when the question is straightforward and unambiguous, minimizing the risk of varied interpretation [68]. Moreover, single-item measures are widely recognized and commonly used within the health care domain [60,69,70], further supporting their validity in this study.

### Outcome Variable: Cancer Genetic Testing

The main outcome variable “Had cancer genetic test” is a dichotomous variable and coded “1” for an individual who had a genetic test for cancer, otherwise “0.” In our data, 142 respondents said they had cancer genetic testing; this represents 8,368,022 in the population when using survey weights. In total, 2805 respondents said they never had cancer genetic testing, which represents 191,142,974 in the population when using survey weights.

We effectively mitigated the potential concern of skewed outcome variables through the application of survey weights. Survey weights are designed to adjust for the complex survey design, including oversampling and nonresponse, ensuring that the results are representative of the target population. Studies have shown that when survey weights are appropriately applied, they can correct for biases introduced by skewed distributions in outcome variables, leading to more accurate and generalizable results [71].

### Primary Independent Variables: Cancer Caregiver and Online Social Support

Caregiver cancer is coded as ‘1’ if the individual is caring for or making medical decisions for someone with cancer, and ‘0’ otherwise. Online social support is coded as ‘2’ for individuals who both share health information on social networking sites and participate in an online forum or support group related to similar health or medical issues. If they engage in only one of these activities, it is coded as ‘1,’ and if neither activity is present, it is coded as ‘0.’

### Support Independent Variables: Health Belief Model

Perceived susceptibility is coded as “1” if first- or second-degree biological relatives had cancer, otherwise “0.” Perceived severity ranges from “1” to “5” for respondents’ general health, varying from “Excellent” to “Poor.” Cues to action are coded as “1” if respondents have heard of cancer genetic testing, otherwise “0.”

The perceived benefit was obtained by summing respondents’ answers to 3 survey questions: How important is knowing a person’s genetic information for preventing cancer? How important is knowing a person’s genetic information for detecting cancer? and How important is knowing a person’s genetic information for treating cancer. These questions have 4 options from “Not at all” to “Very.” Several papers have pointed to income as a potential barrier to preventative health care [72,73]. Hence, we consider income as a proxy for perceived barrier, and it has 5 categories “1” to “5.” Less than US $20,000 is coded as “1” while US $75,000 or more is coded as “5.”

### Moderating Variable: Gender

Females were coded as “1” and male as “0.”

### Control Variables

Race, education, marital status, insurance, and age were used as controls in the model. Whites were coded as “1” and non-Whites were coded as “0.” Education of more than high school was coded “1” for individuals, otherwise “0.” For married individuals, married was coded as “1”, or else “0.” Insured individuals were coded as “1” or else “0.” We used the natural log of “Age” in our model as “Age” varies from 18 to 102 years.

### Statistical Analysis

This study uses survey weights to report national estimates. We used survey-weighted generalized linear models to test the hypotheses in R using the “survey” package. We used HINTS-supplied survey weights using jackknife variance estimation techniques to account for the complex HINTS sampling design and to calculate nationally representative estimates [74]. Since we used publicly available deidentified data, the Institutional Review Board review was exempted.

### Common Method Variance

Data collected with a self-reported single survey may suffer from common method variance (CMV), which hampers the relationship between the variables [75]. To check if our data are suffering from CMV, we used the marker variable technique [76]. A marker variable is a variable that is theoretically unrelated to one or more of the principal variables measured in the study and typically should have a low correlation with the variables of interest.

The theoretically unrelated construct “UnderstandOnlineMedRec” (UOM) was used as a marker variable. The correlation between the marker variable UOM and other principal variables is very low, indicating that CMV is not a problem. The UOM for perceived susceptiblity was 0.03, that for perceived severity was 0.18, that for perceived benefit was −0.03, that for cues to action was −0.21, that for caregiving cancer was 0.02, and that for online social support was 0.02.

### Ethical Considerations

The HINTS 5 survey, conducted with the general population, underwent expedited review and received approval from the Westat Institutional Review Board on March 28, 2016 (project no. 6048.14). In addition, on April 25, 2016, the National Institutes of Health Office of Human Subjects Research determined that the survey did not involve human subjects research, providing an exemption (exempt no. 13204) [77]. This analysis used deidentified, publicly available data from the HINTS, which did not constitute human subjects research as defined by 45 CFR 46.102 and, therefore, did not require IRB review. The original consent and IRB approval cover secondary analysis without the need for additional consent. No compensation was provided for participation.

## Results

### Study Population Characteristics

The data include 3865 civilian, noninstitutionalized US adults aged 18 or older. After filtering for valid responses to the question about genetic testing for high-risk cancer, our final sample consisted of 2947 respondents, representing 199,510,996 US adults. Descriptive statistics of the survey respondents are shown in Table 2 with survey weights applied.

**Table 2.** Descriptive statistics.

Demographic characteristics	Sample size, n (%)	With survey weights
	2947 (100)	199,510,996
Gender		
Male	1126 (38.21)	91,565,888
Female	1623 (55.07)	98,228,842
Race		
White	2192 (74.38)	151,626,358
Non-White	594 (20.16)	36,607,304
Education		
Up to high school	597 (20.26)	50,563,767
More than high school	2278 (77.3)	145,310,461
Insured		
Yes	2775 (97.35)	180,753,226
No	172 (2.65)	18,757,770
Married		
Yes	1434 (48.66)	94,007,748
No	1443 (48.96)	101,703,046
Income		
Less than US $20,000	395 (13.4)	23,749,725
US $20,000 to < US $35,000	312 (10.59)	17,936,144
US $35,000 to <US $50,000	344 (11.67)	21,823,964
US $50,000 to < US $75,000	488 (16.56)	35,145,330
US $75,000 or more	1153 (39.12)	87,555,797
Age, years		
Range	18-102	18-102
Mean (SD)	55.34 (16.65)	47.17 (17.26)

### Multivariable Logistic Regression Analysis

In Table 3, we present the results from the multivariate survey-weighted generalized linear model that provide significant insights into the factors influencing individuals’ decisions to undergo genetic testing for cancer.

**Table 3.** Regression results.

Hypothesis and variables	Estimate	SE	*t* value	Pr(>|t|)	Significant
H1a					
Perceived susceptibility	−0.70805	0.76458	−0.926	0.3616	No
H1b					
Perceived susceptibility: female	2.53956	1.15116	2.206	0.0349	Yes
H2a					
Perceived severity	−0.58773	0.38515	−1.526	0.1372	No
H2b					
Perceived severity: female	0.94366	0.43795	2.155	0.0391	Yes
H3					
Perceived benefit	0.18903	0.08685	2.176	0.0373	Yes
H4					
Income 2	−1.11782	0.68477	−1.632	0.1127	No
Income 3	−0.17123	0.64841	−0.264	0.7935	No
Income 4	0.44008	0.59109	0.745	0.4622	No
Income 5	0.05962	0.55642	0.107	0.9154	No
H5					
Cues to action	2.85722	0.55672	5.132	1.47E-05	Yes
H6					
Caregiving cancer	1.25257	0.60046	2.086	0.0453	Yes
H7					
Online social support	0.47306	0.23093	2.049	0.0491	Yes
Female	−3.48317	1.46416	−2.379	0.0237	Yes
Married	−0.1569	0.30504	−0.514	0.6106	No
LogAge	1.17877	0.43007	2.741	0.0101	Yes
White	−0.07895	0.43618	−0.181	0.8575	No
HighSchoolMore	−0.76716	0.35812	−2.142	0.0401	Yes
Insurance	2.18795	1.21807	1.796	0.0822	No

The analysis did not identify a significant direct relationship between perceived susceptibility and the decision to undergo genetic testing (*β*=−0.70805, *P*=.36), leading to the rejection of hypothesis H1a. However, gender was found to be a significant moderator in this relationship. Specifically, women who perceive themselves as susceptible to cancer are significantly more likely to pursue genetic testing (*β*=2.53956, *P*=.03), providing support for hypothesis H1b.

Similarly, no significant direct association was observed between perceived severity and the decision to undergo genetic testing (*β*=−.58773, *P*=.13), leading to the rejection of hypothesis H2a. However, gender again played a moderating role in this relationship. Women were more likely than men to opt for genetic testing when they perceived cancer as a severe threat (*β*=.94366, *P*=.03), supporting hypothesis H2b.

Beyond gender effects, the findings indicate that individuals who perceive greater benefits from genetic testing are more inclined to undergo testing (*β*=.18903, *P*=.03), confirming hypothesis H3. The expected relationship between perceived barriers—represented by income in this study—and genetic testing decisions (hypothesis H4) was not supported (*β*=−1.11782,−0.17123, 0.44008, 0.05962, *P*>.05).

Cues to action emerged as a significant predictor of genetic testing decisions, with greater exposure to such cues being strongly associated with an increased likelihood of pursuing genetic testing (*β*=2.85722, *P*<.001), supporting hypothesis H5. Additionally, individuals who serve as caregivers for patients with cancer were found to be significantly more likely to engage in genetic testing themselves (*β*=1.25257, *P*=.04), confirming hypothesis H6. Lastly, participation in online social health groups was positively associated with the likelihood of undergoing genetic testing (*β*=.47306, *P*=.04), supporting hypothesis H7.

## Discussion

### Summary of Findings

In line with previous research [78,79], our findings indicate that perceived susceptibility alone may not be enough to motivate individuals to undergo genetic testing. This suggests that additional cues to action or contextual influences may play a crucial role in shaping decision-making.

Moreover, the results show that perceived susceptibility has a stronger effect on genetic testing behavior among women compared to men. One possible explanation is that women may be more sensitive to health risks, particularly hereditary cancers such as breast and ovarian cancer, making them more likely to act on their perceived vulnerability.

This finding is consistent with existing literature [80,81], which suggests that while perceived severity is an important factor, it may not be a sufficient motivator for health-related behaviors without the presence of additional reinforcing elements.

The results also emphasize the importance of accounting for gender differences in health risk perceptions and decision-making. Women’s stronger response to perceived severity may be attributed to their heightened awareness of specific cancer risks and a greater tendency to engage in proactive health behaviors.

Additionally, our findings reinforce a core principle of the HBM, which asserts that perceived benefits are a key driver of health-related actions.

Results further suggest that traditional barriers, such as cost, are being alleviated through evolving health care policies and financial assistance programs [82,83]. Measures such as subsidized testing, reduced out-of-pocket expenses, and insurance coverage for preventive screenings have helped minimize these obstacles [84].

Cues to action emerged as a significant predictor of genetic testing decisions. One explanation for this finding is the changing health care landscape, particularly in preventative care and personalized medicine. Many providers now recognize the value of genetic testing for early risk identification, prompting efforts to reduce financial and logistical barriers [85]. Major insurers increasingly cover preventative genetic testing, reducing costs for individuals [86]. Additionally, online support groups play a role by informing individuals about available financial assistance and providing emotional support, reducing psychological barriers like fear or anxiety about test results [87]. These developments suggest that the traditional “perceived barriers” construct in the HBM may not be relevant in the context of genetic cancer testing.

Another important result from the study is the role of caregiving in influencing decisions to undergo genetic testing. This finding underscores the role of online communities in promoting health-related behaviors by providing access to information, emotional support, and shared experiences. These groups act as cues to action by reducing uncertainty and raising awareness about the benefits of genetic testing, empowering individuals to make more informed health care decisions, especially in preventative care contexts.

### Theoretical Contributions

Our study extends the prior body of research on HBM by examining factors that influence an individual’s decision to undergo cancer genetic testing and incorporating variables that have not been widely explored in prior research. One of the key theoretical advancements of this study is the evidence supporting the moderating role of gender in the relationship between perceived susceptibility, perceived severity, and health behavior change. Our results demonstrate that women are significantly more likely to undergo genetic testing when they perceive higher susceptibility or severity of cancer, thus supporting the hypotheses related to gender moderation. Gender disparity in health decision-making behavior is rooted in cultural, psychological, or social factors that make women more responsive to health risks, particularly those related to cancer, such as breast and ovarian cancer. These findings align with prior research indicating that women are generally more responsive to health risks, particularly in the context of cancer (eg, breast and ovarian cancers), where early detection and preventive measures are critical [35,88]. This gender-specific behavior underscores the need for health interventions that are tailored to reflect differences in health perceptions and behaviors between men and women.

This study extends the HBM by incorporating caregiving as a variable that influences health behaviors. Our results indicate that individuals who are caregivers to patients with cancer are significantly more likely to engage in cancer genetic testing. Caregivers, who are often emotionally and practically involved in managing the health of others, are more attuned to genetic risks and motivated to take preventive action for their own health [89]. This finding suggests that future interventions could target caregivers specifically, providing them with information about the benefits of genetic testing and encouraging preventive health behaviors.

Furthermore, the study highlights the growing importance of digital communities, such as online social health groups, in shaping health behaviors. Our analysis shows that participation in these groups is positively associated with the likelihood of undergoing genetic testing. This finding is consistent with existing literature that points to the influence of social networks on health behavior change [90,91] and the growing importance of digital communities in shaping health behaviors. Online platforms can serve as a source of information, support, and motivation for individuals contemplating health-related decisions. In online platforms, individuals can share experiences, advice, and support, building virtual environments that encourage proactive health actions like genetic testing. As such, health practitioners and policy makers could leverage the power of these digital communities to enhance awareness and promote the benefits of genetic testing and other preventive health measures.

### Practical Implications

This study’s findings have several practical implications for health care providers and public health campaigns. Our results support the importance of perceived benefits in the decision to get genetic testing. Efforts to promote cancer genetic testing should focus on clearly communicating the benefits of early detection, such as the ability to develop personalized cancer treatment plans using information from genetic testing. Early diagnosis significantly improves cancer outcomes, particularly when care is provided at the earliest possible stage [92]. Especially with cancer genetic tests, there is immense potential to facilitate early detection and personalized treatment strategies [93]. Our results also support the importance of cues to action in the decision to get genetic testing. Timely and effective communication can motivate individuals to take proactive steps in their health care journey. However, the success of these interventions depends on individuals’ willingness to engage in preventive health behaviors. Public health officials can pursue changes to health care policy that will incentivize individuals to be proactive about their health and get genetic cancer screenings. Financial incentives have proven to be effective in encouraging individuals to get screenings [94]. Employers already reduce individual monthly insurance premiums by requiring employees to get regular health screenings [95]. Insurance providers can simply add free or reduced-cost genetic cancer screening in the included health care screenings, thus implicitly encouraging and supporting individuals to get screened.

The moderating role of gender highlights the need for improved public health campaigns directed towards men. The Center for Disease Control highlights that “men have higher rates of getting and dying from cancer than women” [96]. There is a lack of online social discourse on genetic testing, which may be the reason for lower male engagement with genetic testing [97]. To address this concern, there has been a notable increase in the number of public health campaigns encouraging men to get screening tests. Recent research has examined how to leverage social media to bring public awareness to the value of genetic testing for prostate cancer [98]. Further efforts in this area could focus on developing messaging on the benefits of early detection using gender-coded language to specifically target men. To improve online social discourse on cancer genetic testing [97], public health organizations can post informative messages on X (formerly Twitter) to encourage discussion of genetic testing, create and participate in regional Facebook groups and pages to develop a community of men interested in genetic testing, and share Facebook and YouTube videos to dispel concerns and address any misconceptions regarding genetic testing. Additionally, our research found the role of caregivers is particularly noteworthy. Our results indicate that caregivers are more likely to seek genetic testing, potentially due to their heightened awareness of cancer risk factors through their caregiving experience [98]. Health care providers should recognize caregivers as a key demographic for targeted interventions. By educating caregivers on the benefits of genetic testing, both for themselves and their families, health care professionals can increase the uptake of this preventive measure. Caregivers, often deeply involved in health care decisions, could also serve as advocates for genetic testing within their broader social networks, further amplifying the reach of these interventions.

In addition, the positive association between online social health group participation and genetic testing uptake suggests that digital platforms can be effective tools for health promotion. These platforms, where individuals can share experiences and seek advice, provide a valuable avenue for disseminating information about the benefits of genetic testing. Public health campaigns that leverage social media and online communities could encourage greater awareness and engagement with preventive health behaviors [99]. Engaging individuals in these groups may also help to reduce the stigma or fear surrounding genetic testing, ultimately facilitating behavior change.

### Limitations and Future Research

Despite its contributions, this study is not without limitations. First, the research relied on secondary data, which constrained the analysis to available variables. The use of secondary data limits the flexibility to explore unmeasured constructs that may further elucidate the decision-making process surrounding genetic testing. Furthermore, we used the HINTS data that were collected in 2020, and hence, it may not fully capture the most recent trends and developments. Future research could consider incorporating primary data collection methods to include more contemporary variables and insights reflective of the current landscape.

Though our study examines the role of caregiving and participation in online support groups in influencing the genetic testing decision, it is important to acknowledge certain limitations that may reduce the potential positive impact of these factors. For instance, barriers such as limited digital literacy, unequal access to the Internet, and socioeconomic disparities can hinder individuals from fully benefiting from online health resources and support networks. These barriers may be particularly pronounced in underserved or marginalized populations, where individuals may lack the necessary tools or knowledge to engage in digital health activities effectively. Future research could address these limitations by exploring how cultural contexts, including beliefs, norms, and values, shape the genetic testing decision. Examining how different communities perceive genetic testing and their access to digital resources can provide more nuanced insights into reducing disparities and improving health outcomes across diverse populations.

### Conclusions

This study explores the factors influencing the decisions to undergo cancer genetic testing in the US population. Our findings emphasize the significant roles of perceived benefits, cues to action, caregiving for patients with cancer, and participation in social health groups in motivating genetic testing. Additionally, gender moderates the relationship between genetic testing and both perceived susceptibility and severity of cancer risk. Given the hereditary nature of cancer, increasing awareness of genetic testing benefits is essential for promoting preventive health behaviors.

The study contributes to both theory and practice. Theoretically, it extends the HBM by incorporating cancer-specific constructs and highlighting the role of information systems in health decision-making. Practically, it offers actionable insights on how tailored education and social support can foster proactive health behaviors, particularly among caregivers and social health communities. Targeted campaigns, especially within online support groups or aimed at men, can further promote early detection. Future research can explore these strategies to increase the adoption of genetic testing for cancer.

## Supplementary material

10.2196/67650 Multimedia Appendix 1 Operationalization of Constructs (source HINTS 5 cycle 4).

## Abbreviations

CMV: common method variance
HBM: health belief model
HINTS: Health Information National Trends Survey
UOM: UnderstandOnlineMedRec
