# Supplementary material for: The Role of Online Support, Caregiving, and Gender in Preventative Cancer Genetic Testing Participation: Cross-Sectional Study From a National Study
Source: JMIR Cancer. 2025 Jun 4;11:e67650. doi: 10.2196/67650 (PMC12157966; doi:10.2196/67650)
Supplement: Multimedia Appendix 1 [file cancer-v11-e67650-s001.docx]

# Multimedia Appendix 1: Operationalization of Constructs (source HINTS 5 cycle 4)

| **Construct** | **Question** | **Scale*** | **Coding*** |
| --- | --- | --- | --- |
| Had Genetic Testing | Have you ever had High-risk cancer genetic tests? | Yes or No | 0: No  1: Yes |
| Perceived Susceptibility | Have any of your first- or second-degree biological relatives (parents, brothers and sisters, children, grandparents, aunts and uncles, nieces and nephews) ever had cancer? | 3: Not Sure  2: No  1: Yes | 0: No or Not Sure  1: Yes |
| Perceived Severity | In general, would you say your health is. | 5: Excellent  4: Very good  3: Good  2: Fair  1: Poor | 1: Excellent  2: Very good  3: Good  4: Fair  5: Poor |
| Perceived Benefit | How important is knowing a person’s genetic information for preventing cancer? | 4: Not at all  3: A little  2: Some  1: A lot | 1: Not at all  2: A little  3: Some  4: A lot  The perceived benefit is the sum of the values and varying from 3 to 12. |
|  | How important is knowing a person’s genetic information for detecting cancer? |  |  |
|  | How important is knowing a person’s genetic information for treating cancer? |  |  |
| Perceived Barrier (Income) | What is your {combined} annual household income? | 5: $75,000 or More  4: $50,000 to < $75,000  3: $35,000 to < $50,000  2: $20,000 to < $35,000  1: less than$20,0000 | 5: $75,000 or More  4: $50,000 to < $75,000  3: $35,000 to < $50,000  2: $20,000 to < $35,000  1: less than$20,0000 |
| Cues to Action | Have you heard of High-risk cancer genetic testing? | Yes or No | 0: No  1: Yes |
| Caregiving to Cancer Patient | Have you provided care for a person with cancer? | Yes or No | 0: No  1: Yes |
| Participation in online social health group | Share health information on social networking sites, such as Facebook or Twitter | Yes or No | For each question, respondents answered ‘Yes’ (coded as 1) or ‘No’ (coded as 0). The Participation in online social health group is the sum of the values and varying from 0 to 2. |
|  | Participate in an online forum or support group for people with a similar health or medical issue |  |  |
| Female | On your original birth certificate, were you listed as male or female? | Male or Female | 0: Male  1: Female |
| Age | What is your age? |  |  |
| Education | What is the highest grade or level of schooling you completed? |  | 0: Up to High school 1: More than High school |
| Race | What is your race? |  | 0: Non-White  1: White |
| Married | What is your marital status? |  | 0: Non-Married  1: Married |
| Insurance | Are you currently covered by any health insurance or health coverage plans? |  | 0: No  1: Yes |
| UnderstandOnlineMedRec | How easy or difficult was it to understand the health information in your online medical record? | 1: Very easy  2: Somewhat easy  3: Somewhat difficult  4: Very difficult | 1: Very easy  2: Somewhat easy  3: Somewhat difficult  4: Very difficult |
| Scale: it represents how questions’ option were provided in the survey.  Coding: It represents how these options were used in this analysis. | | | |
